# Birth weight and the risk of atrial fibrillation in whites and African Americans: the Atherosclerosis Risk In Communities (ARIC) study

**DOI:** 10.1186/1471-2261-14-69

**Published:** 2014-05-26

**Authors:** Sherifat O Lawani, Ellen W Demerath, Faye L Lopez, Elsayed Z Soliman, Rachel R Huxley, Kathryn M Rose, Alvaro Alonso

**Affiliations:** 1 Division of Epidemiology and Community Health, School of Public Health, University of Minnesota, 1300 S 2nd St, Minneapolis, MN, USA; 2 Epidemiological Cardiology Research Center (EPICARE), Wake Forest School of Medicine, Winston-Salem, NC, USA; 3 School of Population Health, University of Queensland, Brisbane, Queensland, Australia; 4 Department of Epidemiology, University of North Carolina, Chapel Hill, NC, USA, and Social and Scientific Systems, Inc, Durham, NC, USA

**Keywords:** Atrial fibrillation, Birth weight, Race, Sex

## Abstract

**Background:**

Low birth weight (LBW) has been associated with an increased risk of cardiovascular disease (CVD). A previous study, however, found higher risk of atrial fibrillation (AF) in individuals with higher birth weight (BW). To further understand this apparent paradox, we examined the relationship between AF and BW in the Atherosclerosis Risk in Communities (ARIC) cohort.

**Methods:**

The analysis included 10,132 individuals free of AF at baseline (1996–1998), who provided BW information, were not born premature, and were not a twin. Self-reported BW was categorized as low (<2.5 kg), medium (2.5-4 kg), and high (>4.0 kg). AF incidence was ascertained from hospital discharge codes and death certificates. We used multivariable Cox proportional hazard models to determine the hazard ratios (HR) and 95% confidence intervals (CI) of AF across BW groups.

**Results:**

During an average follow-up of 10.3 years, we identified 882 incident AF cases. LBW was associated with higher risk of AF. Compared to individuals in the medium BW category, the HR (95% CI) of AF was 1.33 (0.99, 1.78) for LBW and 1.00 (0.81, 1.24) for high BW after adjusting for sociodemographic variables (p for trend = 0.29). Additional adjustment for CVD risk factors did not attenuate the associations (HR 1.42, 95% CI 1.06, 1.90 for LBW and HR 0.86, 95% CI 0.69-1.07 for high BW, compared to medium BW, p for trend = 0.01).

**Conclusion:**

LBW was associated with a higher risk of AF. This association was independent of known predictors of AF and is consistent with that observed for other cardiovascular diseases.

## Background

Atrial fibrillation (AF) is the most common sustained cardiac arrhythmia encountered in clinical practice [1]. AF affects more than 2 million Americans [2], increases the risk of heart failure, stroke, myocardial infarction, and overall mortality [3-6], and contributes significantly to healthcare costs [7].

The developmental origins of health and adult disease postulates that in response to a suboptimal intrauterine environment the fetus employs numerous survival mechanisms, including growth restriction, at the expense of an increased susceptibility to a wide range of chronic disease in adulthood. Birth weight (BW) is frequently used as a proxy for impaired fetal growth *in lieu* of more precise data on fetal growth trajectory. Numerous studies have suggested that low BW (LBW) is related to an increased propensity towards the development of cardiovascular [8,9], metabolic, and endocrine abnormalities in later life [10].

Few data exist regarding the potential relationship between BW and subsequent risk of AF. LBW has been associated with the increased risk of development of hypertension later in life [11], an established risk factor of AF. However, the only published study that explored the impact of BW on risk of AF in adulthood, based on the Women’s Health Study, found that higher BW was associated with an increased risk of AF, rather than decreased risk as might be expected given the fetal origins hypothesis [12].

The incidence of AF is significantly lower in African Americans compared to whites despite the increased prevalence of risk factors of AF in African Americans, such as hypertension or obesity [13-15]. In addition, the prevalence of LBW is higher among African Americans than whites [16]. Assessing whether black-white differences in BW could explain the paradoxical racial disparity in AF risk merits attention.

Therefore, the aim of this study was to assess the relationship between BW and AF risk, including assessment of effect modification by gender and race, using data collected in the Atherosclerosis Risk in Communities (ARIC) cohort.

## Methods

### Study population

The ARIC study is an ongoing prospective cohort in four communities in the US: Forsyth County, North Carolina; Washington County, Maryland; Minneapolis suburbs, Minnesota; and Jackson, Mississippi [17]. Participants in the cohort were randomly selected from a defined population of age 45–64. At baseline (1987–1989), 15,792 participants were recruited and received medical examinations. Three follow-up examinations were performed every three years, and a fourth follow-up exam was conducted in 2011–2013. In addition, study participants receive annual phone follow-up calls to get updates on health and vital status. The ARIC study is performed in accordance with the Declaration of Helsinki and has been approved by Institutional Review Boards (IRB) at all participating institutions: University of North Carolina at Chapel Hill IRB, Johns Hopkins University IRB, University of Mississippi Medical Center IRB, and University of Minnesota IRB. Study participants provided written informed consent at all study visits.

For this study we included only whites and African Americans who participated in ARIC visit 4 (1996–98), when BW was assessed. Individuals with missing information on BW or any other covariate of interest, reported cases of prematurity, twins, and individuals with prevalent AF at visit 4 were excluded. Table 1 shows the exclusion criteria with the resultant 10,132 observations used in the analysis.

**Table 1:** Number of excluded participants according to different exclusion criteria, ARIC study, 1996-1998

**Exclusion criteria**	**Individuals excluded**	**Participants**
Initial cohort in 1987-89		15,792
Attended visit 4 (1996–98)	4,136	11,656
Prevalent AF at visit 4	298	11,358
Low or bad quality ECGs at baseline	175	11,183
Race other than white or African American	67	11,116
Missing Covariates	151	10,965
Twin gestation or premature infant	490	10,475
Missing BW	343	10,132

### Study measures

#### Birth weight

At the visit 4 exam (1996–98), participants were asked to recall their exact BW in pounds and ounces, and those who could not recall their exact BW were asked to categorize it into one of three categories: low (<5.5 lbs/<2.5 kg.), medium (5.5–9.0 lbs./2.5-4.0 kg) or high (>9.0 lbs./>4.0 kg). Actual reported weights were grouped using these same categories. This three-level ordinal variable was used in all analyses. Participants reporting exact BW were more likely to be white and female [18]. In the ARIC cohort, categories of self-reported exact BW followed a similar distribution to BW in the general US population [18]. Studies in other populations suggest that self-reported birth weight has moderate to good validity [19].

#### AF ascertainment

Data on AF was obtained from 12-lead ECGs performed in study exams, from ICD-9 codes from hospitalization discharges (427.31, 427.32) or from death certificates if AF was listed as any cause of death (ICD-9 427.3 or ICD-10 I48) [15]. More than 90% of AF cases were identified from hospital discharges. In the present analysis, we considered incident AF as any first occurrence of AF between visit 4 and December 31, 2008; after visit 4, all AF cases were identified from hospital discharge codes and death certificates.

#### Other variables

Other covariates were defined based on information collected at visit 4 (with the exception of education level and income, which were collected at visit 1). Questionnaires provided information on age, gender, educational level, income, cigarette smoking, alcohol intake, and use of hypertensive medication. Blood pressure, height and weight were measured at the study visit. Body mass index (BMI) was computed as weight in kilograms divided by height in meters squared. Diabetes was defined as fasting serum glucose ≥126 mg/dL, use of anti-diabetic medication, non-fasting serum glucose ≥200 mg/dL, or a self-reported diagnosis of diabetes. Prevalent MI and heart failure were defined as previously described [20,21].

### Statistical analysis

**Figure 1:**
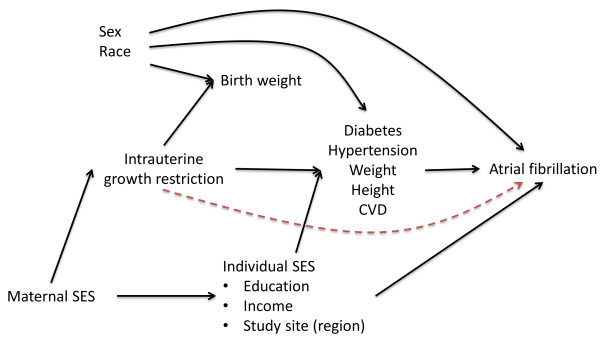
**Causal model for the association of birth weight, as a proxy for intrauterine growth restriction, with the incidence of atrial fibrillation.** The red-dashed line represents a hypothetical direct effect of intrauterine growth retardation on atrial fibrillation incidence not mediated through known cardiovascular risk factors. CVD: Cardiovascular disease. SES: Socioeconomic status.

The causal model guiding our selection of covariates for multivariable analysis is presented in Figure 1. A brief justification for this model follows. Our main question of interest is whether intrauterine growth retardation, assessed with BW as a proxy, leads to AF. Based on existing literature, this association could be mediated by an increase in the risk of cardiovascular risk factors (diabetes, hypertension, obesity, etc.) and cardiovascular disease [8-10], which are well-established risk factors for AF [22]. Male gender and white race are associated with higher birth weight in our sample (Table 1) and are also associated with AF risk [15], therefore being considered confounders. Maternal socioeconomic status (SES), through a range of mechanisms, is associated with intrauterine growth retardation and, through its impact on an individual’s SES, can affect the future risk of AF. Since we do not have information on maternal SES, we used individual SES as a proxy, including education, income, and study site as potential confounders. Finally, we considered all other cardiovascular risk factors as variables possibly in the causal pathway between intrauterine growth retardation/BW and AF risk. Estimation of the total effect of birth weight on AF should not adjust for those variables; however, a model adjusting for those variables could be used to test whether any association between BW and AF is mediated through changes in cardiovascular risk factors or through other, not described, pathways (represented by the red dashed line in Figure 1), provided that some assumptions are met. These assumption include the absence of uncontrolled confounding between the exposure and the outcome, absence of uncontrolled confounding between the mediator and the outcome, and no effect measure modification between the exposure and the mediator in their association with the outcome [23].

In all analyses, we categorized BW into low (<2.5 kg), medium (2.5 - 4.0 kg), and high (>4.0 kg). The association between categories of BW and incidence of AF was estimated using hazard ratios (HR) and 95% confidence intervals (CI) obtained from Cox proportional hazards models, using medium BW as the reference category. An initial analysis was conducted on the entire sample, followed by race and gender-stratified analyses. Time of follow-up was defined as number of days between visit 4 and incident AF, death, lost during follow-up, or December 31, 2008, whichever occurred earlier. We conducted initial analysis adjusted for potential confounders: Model 1 adjusted for age, race and gender. Model 2 adjusted additionally for socioeconomic factors (income and education) and study center. Variables in models 1 and 2 can be considered confounders since they may be determinants of BW and are also risk factors for AF. Finally, we ran an additional model 3 that included cardiovascular risk factors as potential mediators, i.e. in the causal pathway, of the association between BW and AF risk (diabetes, systolic blood pressure (SBP), hypertension medications, smoking, height, BMI, prevalent myocardial infarction, and heart failure). Based on our assumptions about causal relationships, this model specifically tests whether a direct effect of BW on AF risk exists independently of these mediators (Figure 1). Assumptions for this test include: (1) no uncontrolled confounding between the exposure (BW) and the outcome (AF), (2) no uncontrolled confounding between the mediators (cardiovascular risk factors) and the outcome (AF), and (3) no effect measure modification between the exposure and the mediators [23]. We considered the first two assumptions to hold because of our adjustment for predictors of BW, AF, and cardiovascular risk factors. We did not find significant effect measure modification between BW and prevalence of cardiovascular risk factors regarding the association with AF (data not shown), considering the third assumption to hold.

Pre-specified analyses testing for interactions by race and gender were performed including multiplicative terms in the models and conducting race- and gender-stratified analyses. Test for trends were conducted by including BW category in the models as an ordinal variable. We conducted two sensitivity analyses, first including only those participants who reported their exact BW in pounds and ounces, and second starting follow-up at ARIC visit 1, instead of visit 4, and including all incident cases from visit 1. All analyses were conducted with SAS version 9.2 (SAS Institute Inc., Cary, NC).

## Results

### Baseline characteristics

At visit 4, the mean age of the 10,132 eligible individuals was 62.7 years (standard deviation, 5.7). Of these, 449 (4%) reported LBW, 1,086 (11%) reported high BW, while the remaining 8,597 (85%) reported medium BW.

Table 2 shows characteristics of the study sample by categories of BW. Approximately 44% of the eligible participants were men. Women and African Americans were more likely to be in the LBW category (P < 0.001).

**Table 2:** Baseline characteristics of study participants by self-reported birth weight categories, ARIC study, 1996-2008

	**Birth weight categories**			
	**Low**	**Medium**	**High**	P value^*^
N (%)	449 (4%)	8,597 (85%)	1,086 (11%)	
Age, years (mean ± SD)	62.7 ± 5.6	62.7 ± 5.7	62.8 ± 5.6	0.89
Male, %	22.1	42.6	59.1	<0.001
African American, %	26.7	21.1	16.3	<0.001
Less than high school degree, %	25.2	18.3	15.8	<0.001
BMI, kg/m^2^ (mean ± SD)	28.5 ± 6.0	28.7 ± 5.5	29.4 ± 5.9	0.001
Height, cm (mean ± SD)	162.6 ± 8.6	168.2 ± 9.0	172.5 ± 9.1	<0.001
Diabetes, %	19.8	15.8	14.6	0.04
SBP, mmHg (mean ± SD)	129.3 ± 19.1	127.4 ± 18.9	125.4 ± 18.0	<0.001
Hypertensive medication use, %	47.4	42.4	39.2	0.01
Current smoker, %	17.8	14.2	17.3	<0.001
Prevalent heart failure, %	1.3	1.3	2.2	0.06
Prevalent myocardial infarction, %	3.3	2.7	2.9	0.69
Incident atrial fibrillation, %	10.9	8.6	9.2	0.21

BMI: Body mass index; SBP: systolic blood pressure.

*P value based on chi square test for categorical variables and analysis of variance for continuous variables.

### Association between BW category and incident AF

During an average 10.3 years of follow-up, we identified 882 incident AF cases. The crude incidence rate of AF in the study population was 8.4 events per 1000 person years. Incidence rates across BW categories were: 10.5 events per 1000 person-years for those with LBW, 8.3 events per 1000 person-years for the medium BW category, and 8.8 events per 1000 person-years for the high BW category (Table 3).

**Table 3:** Hazard ratios and 95% confidence intervals of atrial fibrillation by birth weight category, ARIC study, 1996-2008

	**Birth weight categories**			
	**Low**	**Medium**	**High**	**P for trend**
AF events, N	49	735	98	
Person-years	4,662	88,982	11,095	
Incidence Rate* (95% CI)	10.5 (7.9-13.8)	8.3 (7.7-8.9)	8.8 (7.2-10.7)	
Model 1	1.37 (1.03, 1.84)	1 (ref.)	1.00 (0.81, 1.23)	0.21
Model 2	1.33 (0.99, 1.78)	1 (ref.)	1.00 (0.81, 1.24)	0.29
Model 3	1.42 (1.06, 1.90)	1 (ref.)	0.86 (0.69, 1.07)	0.01

*Incidence rate per 1000 person years.

Model 1: Cox proportional hazards model adjusted for age, race and gender.

Model 2: As model 1, additionally adjusted for study center, income, and education.

Model 3: As model 2, additionally adjusted for diabetes, systolic blood pressure, hypertension medications, smoking, height, body mass index, prevalent. myocardial infarction and heart failure.

In multivariable models, LBW was associated with a higher risk of AF compared to medium BW (Table 3). After adjusting for socio-demographic variables (Model 2), individuals in the LBW category had an approximate 33% increase in AF risk compared to those with medium BW: HR 1.33, 95% CI 0.99, 1.78. High BW, compared to medium BW, was not associated with the risk of AF (HR 1.00, 95% CI 0.81, 1.24). The association remained after controlling for potential mediators (Model 3), suggesting the presence of alternative pathways between BW and AF risk. Results remained essentially unchanged when we started the follow-up at visit 1, including all incident cases occurring afterwards (1131 AF cases; Model 2: HR 1.34, 95% CI 1.03, 1.74 for LBW; HR 1.04, 95% CI 0.86, 1.25 for high BW, compared to medium BW).

An analysis restricted to the 4810 ARIC participants (including 412 AF cases) who reported their exact BW in pounds and ounces provided similar estimates of association. The HRs (95% CI) for low and high BW compared to medium BW were respectively 1.23 (0.85, 1.79) and 0.91 (0.71, 1.16) in models adjusted for sociodemographic variables, and 1.32 (0.91, 1.93) and 0.81 (0.63, 1.04) after additional adjustment for cardiovascular disease risk factors.

### Association between BW and incident AF by race

The interaction term race*BW was not statistically significant (p-value = 0.28). Nonetheless, we conducted race-stratified analysis, as pre-specified in our hypothesis. Table 4 shows the association between BW and AF by race. Among whites, LBW was associated with higher risk of AF, with an increase of 35% in AF risk in models adjusted for sociodemographic characteristics, compared to LBW. The association was less apparent among African Americans, with individuals in the medium BW category showing the lowest risk of AF. African-American race was associated with lower risk of AF compared to white race after adjusting for age, gender, and birth weight category (HR 0.84, 95% CI 0.70-1.00).

**Table 4:** Hazard ratios and 95% confidence intervals of atrial fibrillation by birth weight category, stratified by race, ARIC study, 1996-2008

	**Birth weight categories**			
**Whites (N = 8,022, AF events = 743)**	**Low**	**Medium**	**High**	**P for trend**
AF events, N	38	624	81	
Person-years	3,471	70,183	9,304	
Incidence Rate* (95% CI)	10.9 (7.9-14.9)	8.9 (8.2-9.6)	8.7 (7.0-10.8)	
Model 1	1.35 (0.97, 1.88)	1 (ref.)	0.92 (0.73, 1.16)	0.10
Model 2	1.30 (0.94, 1.81)	1 (ref.)	0.92 (0.73, 1.16)	0.13
Model 3	1.38 (0.99, 1.92)	1 (ref.)	0.79 (0.63, 1.00)	0.007
**African Americans (N = 2,110, AF events = 139)**	**Low**	**Medium**	**High**	
AF events, N	11	111	17	
Person-years	1,191	18,798	1,791	
Incidence Rate* (95% CI)	9.2 (4.9-16.0)	5.9 (4.9-7.1)	9.5 (5.7-14.9)	
Model 1	1.51 (0.81, 2.81)	1 (ref.)	1.64 (0.98, 2.74)	0.55
Model 2	1.49 (0.80, 2.78)	1 (ref.)	1.75 (1.04, 2.93)	0.44
Model 3	1.60 (0.86, 2.99)	1 (ref.)	1.37 (0.80, 2.34)	0.97

*Incidence rate per 1000 person years.

Model 1: Cox proportional hazards model adjusted for age and gender.

Model 2: As model 1, additionally adjusted for study center, income, and education.

Model 3: As model 2, additionally adjusted for diabetes, systolic blood pressure, hypertension medications, smoking, height, body mass index, prevalent. myocardial infarction and heart failure.

### Association between BW category and incident AF by gender

As with race, no significant interaction was observed between gender and BW (p-value = 0.21). The pre-specified analysis showing the association between BW and AF risk by gender is presented in Table 5. LBW showed a strong association with AF risk in men, while in women, no clear evidence of an association between BW and AF risk was present.

**Table 5:** Hazard ratio and 95% confidence intervals of atrial fibrillation by birth weight category, stratified by gender, ARIC study, 1996-2008

	**Birth weight categories**			
**Men (N = 4,403, AF events = 453)**	**Low**	**Medium**	**High**	**P for trend**
AF events, N	19	374	60	
Person-years	982	36,754	6,410	
Incidence Rate* (95% CI)	19.4 (12.0-29.6)	10.2 (9.2-11.2)	9.4 (7.2-12.0)	
Model 1	1.92 (1.21, 3.04)	1 (ref.)	0.92 (0.70, 1.20)	0.07
Model 2	1.91 (1.20, 3.03)	1 (ref.)	0.92 (0.70, 1.20)	0.07
Model 3	1.91 (1.20, 3.04)	1 (ref.)	0.80 (0.61, 1.06)	0.009
**Women (N = 5,729, AF events = 429)**	**Low**	**Medium**	**High**	
AF events, N	30	361	38	
Person-years	3,680	52,227	4,685	
Incidence Rate* (95% CI)	8.2 (5.6-11.5)	6.9 (6.2-7.7)	8.1 (5.8-11.0)	
Model 1	1.17 (0.80, 1.69)	1 (ref.)	1.16 (0.83, 1.62)	0.92
Model 2	1.13 (0.78, 1.64)	1 (ref.)	1.17 (0.83, 1.63)	0.80
Model 3	1.23 (0.84, 1.79)	1 (ref.)	0.97 (0.67, 1.36)	0.51

*Incidence rate per 1000 person years.

Model 1: Cox proportional hazards model adjusted for age and gender.

Model 2: As model 1, additionally adjusted for study center, income, and education.

Model 3: As model 2, additionally adjusted for diabetes, systolic blood pressure, hypertension medications, smoking, height, body mass index, prevalent. myocardial infarction and heart failure.

## Discussion

Our study showed an association between BW and incident AF, with higher risk of AF among individuals with LBW, compared to medium BW. These results are similar to what has been generally observed for the relationship of BW with other cardiovascular diseases [8,9]. The association was more evident in men and whites; BW was a weaker risk factor for AF in women and African Americans. Our results are consistent with the fetal origin hypothesis of coronary heart disease and other developmental theories that associate cardiovascular disease in adulthood with undernutrition in utero [24]. Adverse prenatal factors that may have caused intrauterine growth restriction could also lead to metabolic and organ changes that can predispose affected individuals to chronic disease in adulthood. This suggests that LBW, an indication of possible intrauterine growth restriction, may be a risk factor for developing AF later in life.

LBW has been associated with a higher risk for hypertension and diabetes [10,11]. Reduced fetal growth may result in changes in fetal blood flow or hormonal variations, which can lead to abnormal development of various organs involved in blood pressure control including the kidneys, autonomic nervous system, endocrine glands, and cardiac vasculature. In the kidney, these changes would result in fewer nephrons, potentially leading to poor salt regulation and hypertension [25]. In the pancreas, they could result in a reduced beta cell mass and poor regulation of glucose later in life predisposing one to diabetes. It has been suggested that the higher prevalence of diabetes in individuals with LBW could reflect a selective survival of LBW infants genetically susceptible to diabetes [26]. Studies have shown that obesity, diabetes and hypertension are strong risk factors for developing AF [27,28]. Therefore, the increased risk of AF associated with LBW could be mediated through a higher risk of cardiometabolic risk factors. In our analysis, however, associations remained after adjustment for cardiovascular risk factors, which would suggest that other variables mediate this association.

Results from the ARIC Study contradict those previously reported in the Women’s Health Study, which found a *higher* risk of AF among women with higher BW [12]. Some important differences exist between both studies, though. The Women’s Health Study included only women, mostly white, all of them health professionals. The ARIC study, in contrast, included a biracial population of both genders, with a more diverse educational background. Other differences between studies include the potential different quality of BW information (possibly higher in the Women’s Health Study, since participants were all female and more educated on average), the method for ascertainment of AF, and the availability of potential confounders. Future research in other population should try to clarify this apparent inconsistency.

In race and gender-stratified models, we found that the association of LBW with the risk of incident AF was stronger in men and whites than in women and African Americans, although these differences were not statistically significant. Possible explanations for the gender and race disparity are the differential distribution of BW and different degree of misclassification in BW information across different demographic groups. Of note, the previously reported lower AF risk in African Americans vs whites [15] in the ARIC cohort remained after adjustment for birth weight categories.

### Study limitations and strengths

Several important limitations exist regarding the information on BW. First, about 50% of the participants could not recall their exact BW and, thus, only BW categories could be used (low, medium, high BW). Second, the overall prevalence of LBW in our sample (4%) is lower than expected, suggesting underreporting of LBW [18]. In an analysis including only those with self-reported exact BW, which possibly provided more valid BW information [18], results remained unchanged. Third, the absence of gestational age information also adds to misclassification errors; infants experiencing normal fetal growth but born somewhat earlier are combined with those that are small for gestational age, which suggests intrauterine growth restriction. However, participants were asked if they were born premature, and excluded if the answer was affirmative, thus decreasing the magnitude of this potential misclassification. Similarly, the relatively low number of AF events in the LBW category may compromise the robustness of our results. Another important limitation is that AF was mostly ascertained from hospital discharge codes, which could lead to missing AF events identified in outpatient settings only. We and others have showed previously, however, that this method has adequate validity for epidemiologic studies of AF [15,29]. Finally, BW information was not collected until study participants were in late midlife (average age at baseline 63), which might lead to selection bias if study dropout status or non-participation was associated with BW and AF or AF risk factors. However, we adjusted our analysis for known established risk factors for AF thus reducing this bias [30].

Major strengths of this study include the large sample size, the inclusion of both whites and African Americans, extended follow-up, and availability of information on confounding variables (socioeconomic status) and mediators (anthropometry, cardiovascular risk factors).

## Conclusion

In conclusion, in this biracial cohort study, LBW was associated with an increased risk of incident AF. This association was independent of known predictors of AF such as hypertension, BMI and height. This association may be stronger in men and whites. Given the differences in results from the ARIC Study and the Women’s Health Study, additional research in other diverse populations should be conducted to clarify the relationship between birth weight and AF risk.

The authors thank the staff and participants of the ARIC study for their important contributions.

## Competing interests

The authors have no conflict of interest to disclose related to the present manuscript.

## Authors’ contributions

The study was designed by SOW, EWD, KMR, and AA. Data on AF were collected by EZS and AA. SOW and FLL analyzed the data. SOW and AA wrote the first draft of the manuscript. All authors participated in data interpretation, discussion and preparation of the final manuscript, and read and approved the final manuscript.

## Pre-publication history

The pre-publication history for this paper can be accessed here:

http://www.biomedcentral.com/1471-2261/14/69/prepub
